# Supplementary material for: Epigenetic Transitions and Knotted Solitons in Stretched Chromatin
Source: Sci Rep. 2017 Nov 7;7:14642. doi: 10.1038/s41598-017-13916-w (PMC5676697; doi:10.1038/s41598-017-13916-w)
Supplement: Supplementary file 1 — Supplementary Information [file 41598_2017_13916_MOESM1_ESM.pdf]

Epigenetic Transitions and Knotted Solitons in Stretched Chromatin
Supplementary Material

D. Michieletto[1,*], E. Orlandini[2], D. Marenduzzo[1]
[1] *SUPA, School of Physics and Astronomy, University of Edinburgh, Peter Guthrie Tait Road, Edinburgh, EH9 3FD, UK.* [2] *Dipartimento di Fisica e Astronomia and Sezione INFN, Universitá di Padova, Via Marzolo 8, Padova 35131, Italy*
[*] *corresponding author: davide.michieletto@ed.ac.uk*

TRUNCATED AND SHIFTED LENNARD-JONES

Attractive and repulsive interactions are described by a truncated-and-shifted Lennard-Jones potential which is broadly used in Molecular Dynamics simulations to model short-ranged interactions as it is both, computationally efficient and realistic. In addition to its standard use, we include a "colour-dependence" in order to model like-colour attraction and different-colour repulsion. We do this by choosing a colour-dependence cut-off r_c and as described in Eqs. (1-2) of the main text. The potential is shown in Fig. S1. As one can readily notice from the figure, the potential does not display discontinuities, thanks to the shift at the cut-off point (last two terms in Eq. (1) of the main text).

PHASES OF THE SYSTEM

Typical examples of trajectories for the number of red, blue and grey beads over the course of a simulation near the transition are reported in Fig. S2. The multi-domain phase displays a slow convergence of the system towards an all-red or all-blue situation. Nonetheless, these are not reached within the simulation time and the distribution of magnetisations is thus more uniform than for the "ordered" or "disordered" cases. At the critical point (which occurs at around $f = 2.45 k_B T_L/\sigma$ for $\epsilon = 2 k_B T_L$, one can observe coexistence of ordered and disordered states, as shown in Fig. S3. In that figure, one can observe that the distribution of absolute magnetisation m and radius of gyration R_g both display bimodal shapes, supporting the claim that the transition is abrupt (first order).

Supplementary Movies

- `M1_CO.avi` Movie showing chromatin collapsing into the compact-epigenetically ordered state corresponding to the kymograph in Fig. 2(B).
- `M2_SD.avi` Movie showing chromatin retaining the stretched-epigenetically disordered state corresponding to the kymograph in Fig. 2(A).
- `M3_MD.avi` Movie showing the emergence of a multi-domain configuration due to the simultaneous formation of nucleation points corresponding to the kymograph in Fig. 3(B) and the snapshot in Fig. 3(E).
- `M4_Knot41_1.avi` Movie showing the diffusion of a knotted soliton along stretched chromatin corresponding to the full kymograph in Fig. 4(A).
- `M5_Knot41_2.avi` Movie showing the diffusion of a knotted soliton along stretched chromatin corresponding to a section of the kymograph in Fig. 4(D).

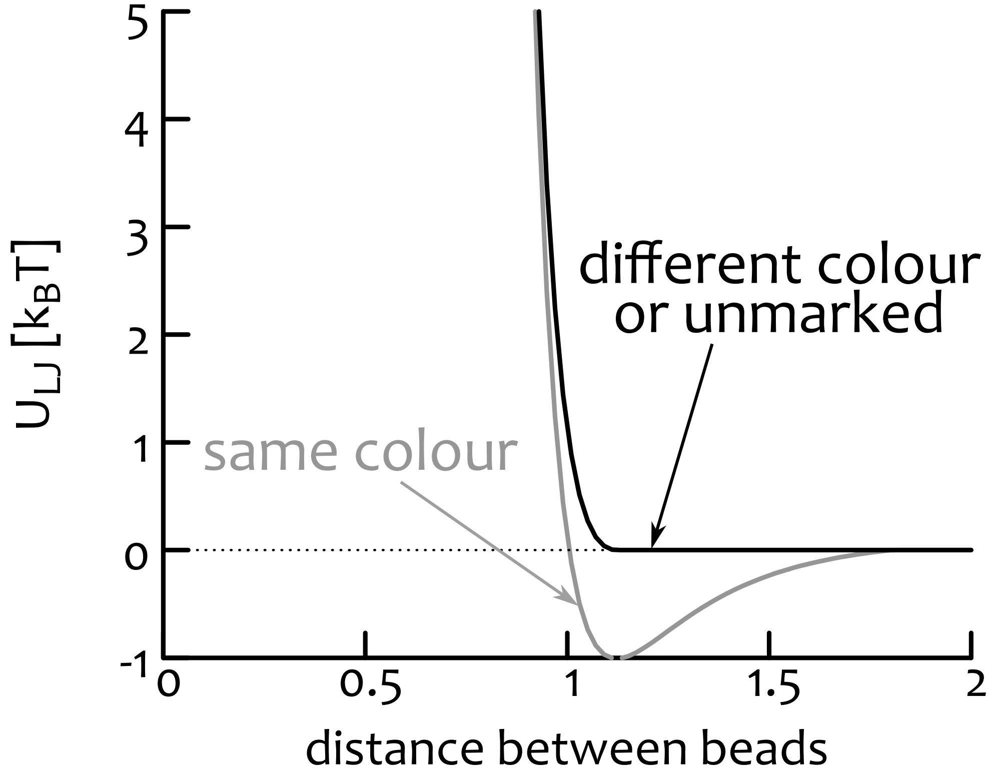

Fig. S **1**. Plot of the truncated and shifted Lennard-Jones (LJ) potential used to model attractive and repulsive interactions between the beads in the simulation. For same colour beads ($q_a = q_b$), the potential displays an attractive (negative) region by setting the cut-off length $x_c^{q_a q_b} = 1.8\sigma$ as described by Eq. 1 of the main text. For purely repulsive interactions (those between differently coloured beads or when at least one of the interacting beads is unmarked) the potential is truncated at its minimum $x_c^{q_a q_b} = 2^{1/6}\sigma$ and shifted so that there remains only its repulsive (positive) part. The normalisation constant $\mathcal{N}$ ensures that the minimum of the potential is at -$k_B T$. The strategy to cut and shift the LJ potential is common in Molecular Dynamics simulations as it is computationally efficient (short-ranged) while remaining representative of two-body interactions.

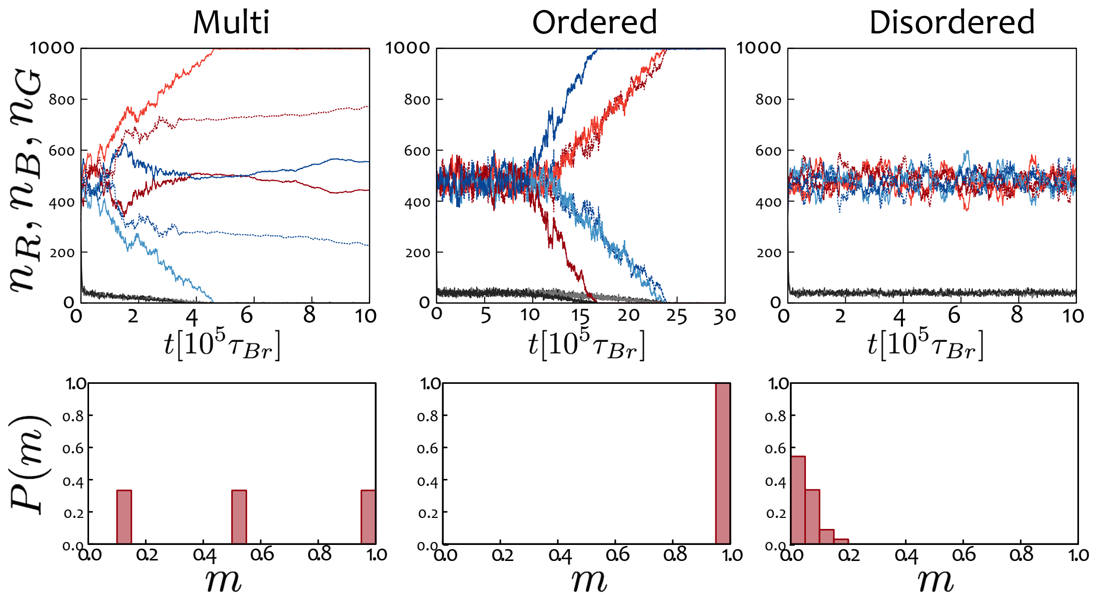

Fig. S **2**. (**Top row**) Plots showing three examples of trajectories per each combination of $\epsilon = 2k_BT_L$ and $f = 2, 2.4$ and 2.5 k_BT_L/σ, respectively. These show the number of red (n_R, shades of red), blue (n_B, shades of blue) and grey (n_G, shades of grey) beads as a function of time. One can notice that in the "multi-domain" phase the number of beads slowly evolve towards an all-red or all-blue states (which is reached in one of the reported examples); the corresponding distribution of absolute magnetisation (**bottom row**) is uniform and not peaked near zero or unity. This suggests that the multi-domain state is long-lived but not stable. Ordered and disordered phases display either symmetry breaking or fluctuation around an equal number of red and blue beads, respectively. The corresponding distributions of magnetisation are peaked at one or zero, respectively.

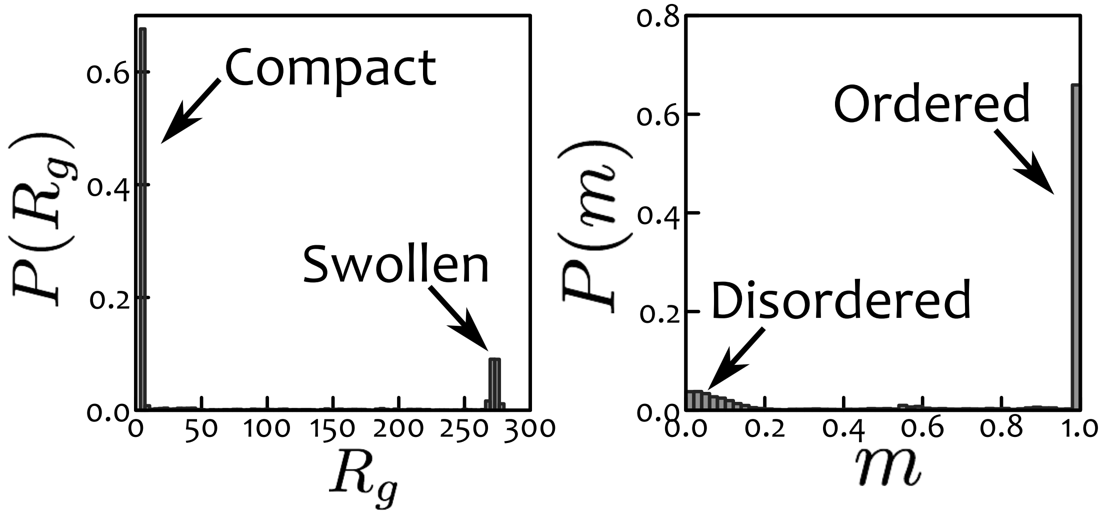

Fig. S **3**. Coexistence of swollen-disordered (large R_g and small m) and compact-ordered (small R_g and large m). Here the combination of parameters is $\epsilon = 2k_BT_L$ and $f = 2.45k_BT_L/\sigma$.
